# Error Fusion of Hybrid Neural Networks for Mechanical Condition Dynamic Prediction

**DOI:** 10.3390/s21124043

**Published:** 2021-06-11

**Authors:** Wentao Zhang, Yucheng Liu, Shaohui Zhang, Tuzhi Long, Jinglun Liang

**Affiliations:** 1 School of Mechanical Engineering, Dongguan University of Technology, Dongguan 523808, China; zhangwentao@dgut.edu.cn (W.Z.); 1910293061@email.szu.edu.cn (Y.L.); 2111804367@mail2.gdut.edu.cn (T.L.); liangjl@dgut.edu.cn (J.L.); 2 College of Mechatronics and Control Engineering, Shenzhen University, Shenzhen 518060, China; 3 School of Automation, Guangdong University of Technology, Guangzhou 510006, China

**Keywords:** mechanical equipment, error fusion of multiple SAEs (EFMSAE), convolutional neural networks (CNN), prediction

## Abstract

It is important for equipment to operate safely and reliably so that the working state of mechanical parts pushes forward an immense influence. Therefore, in order to enhance the dependability and security of mechanical equipment, to accurately predict the changing trend of mechanical components in advance plays a significant role. This paper introduces a novel condition prediction method, named error fusion of hybrid neural networks (EFHNN), by combining the error fusion of multiple sparse auto-encoders with convolutional neural networks for predicting the mechanical condition. First, to improve prediction accuracy, we can use the error fusion of multiple sparse auto-encoders to collect multi-feature information, and obtain a trend curve representing machine condition as well as a threshold line that can indicate the beginning of mechanical failure by computing the square prediction error (*SPE*). Then, convolutional neural networks predict the state of the machine according to the original data when the *SPE* value exceeds the threshold line. It can be seen from this result that the EFHNN method in the prediction of mechanical fault time series is available and superior.

## 1. Introduction

In the context of the current Internet of Things, narrowing the gap between real-time data of enterprises’ factories and business decisions is essential. Predictive maintenance services are used to substitute state-based maintenance services of enterprises [1]. Machine parts will appear in different types of failure significantly, which reduces the machine equipment’s efficiency when it works for a long time. If relevant measures are not taken in time, severe economic losses and safety accidents may occur. Therefore, it is necessary to study the prediction of equipment failure. In recent years, the forecast of mechanical equipment has developed rapidly because it can improve the equipment’s reliability and safety, significantly improve the production efficiency, and reduce maintenance costs through fault prediction of mechanical equipment [2].

Mechanical equipment’s health monitoring has become an essential part of promoting the big data revolution and the development of modern manufacturing systems with the support of industrial Internet and data-driven technology [3]. In recent years, advanced sensing technology and network communication have grown rapidly, which has provided much data for manufacturing systems. Traditional data-driven prediction is based on shallow learning architecture and cannot meet the demand of big data. As an important branch of machine learning, deep learning can extract valuable information from big data and process it to make appropriate decisions, thus achieving rapid development. Deutsch et al. [4] proposed a method based on deep learning to predict the residual life (RUL) of big data rotation components. Sun et al. [5] proposed a method based on deep learning to predict the tool’s working state. Zhao et al. [6] used a local feature-based gated recurrent unit (LFGRU) network to predict the machine state. Shao et al. [7] realized and accelerated the deep learning framework of deep neural network training by using transfer learning to achieve highly accurate machine fault diagnosis. Chen et al. [8] proposed an RNN model based on encoder–decoder structure and attention mechanism, which was used to mine useful degradation information from long historical data and verified by visual attention distribution. Chang et al. [9] studied the iterative gradient convergence of the back-propagation neural network (BNN) algorithm used to predict tool life and analyzed its convergence and stability. Fang et al. [10] conceived the prediction of the jobs remaining time (JRT) based on the production of big data (BD) deep learning (DL) in the manufacturing execution process. By combining time-domain features and frequency-domain features, Ren et al. [11] proposed an integrated deep learning method for collaborative prediction of the remaining service life of multiple bearings. Sun et al. [12] proposed a deep transfer learning (DTL) network based on a sparse autoencoder (SAE) for machine failure prediction.

The literature mentioned above shows that before predicting the workpiece, there are three stages: data processing, feature extraction, and model training. The key part is improving the prediction accuracy because data processing and feature extraction will significantly impact the model’s prediction accuracy. Training data and test data are mostly collected by sensors, such as sensors related to vibration, acceleration, temperature, and air pressure, or combining multiple sensors of the same type to collect real-time working status data of mechanical equipment [13]. The Vibration data belong to the natural time series, the LSTM model has become the primary processing time series method [14]. Wu et al. [15] constructed a new deep long-term short-term memory (DLSTM) model that uses multiple sensor time series signals to accurately predict the remaining service life (RUL). Wu et al. [16] proposed to use the utilizing vanilla LSTM neural network to obtain an excellent remaining useful life (RUL) prediction accuracy. Cai et al. [17] proposed a hybrid information system for tool wear prediction based on a long-term short-term memory network (LSTM). These prediction methods based on deep learning have certain shortcomings while achieving certain results. For example, their prediction performance depends mainly on the hand-made feature design [18]. In the actual data acquisition process, especially in the acquisition of rotating machinery vibration data, often noise interference makes it more difficult to extract data features. As an essential part of deep learning, due to the good ability to extract features, CNN is widely used in data feature extraction. Based on the multi-domain feature fusion method, Huang et al. [19] proposed a new deep convolutional neural network (DCNN) model for tool wear prediction. Wang et al. [20] proposed a hybrid prediction scheme completed by a newly developed deep heterogeneous GRU model and local feature extraction. Duan et al. [21] designed a new deep learning multi-frequency-bands deep convolutional neural network (MFB-DCNN) model, which is used to process big data and monitor tool status. Li et al. [22] proposed a deep learning method for multi-scale feature extraction based on convolutional neural networks for remaining life prediction. Wang et al. [23] proposed a system prediction method for a recurrent convolutional neural network (RCNN) for mechanical RUL prediction. Fu et al. [24] combined convolutional neural networks (CNN) with long-term and short-term memory networks (LSTM) for fan status monitoring. Recently, the EFMSAE-LSTM algorithm (literature [25]) was proposed for bearing condition dynamic monitoring.

At present, the research mainly focuses on the detection, diagnosis, and classification of faults with deep learning algorithms. However, there is less research on the trend of machine faults. The LSTM model algorithm can predict machine failure trends, but it only concerns the long-term sequence information, which will lose global features. On the contrary, based on autonomous learning, the CNN method can extract essential features of the sequence from a global perspective. Both LSTM and CNN need an excellent data input mode, that is, the input data are larger, and the calculation is less efficient. Consulting the literature, it was discovered that Zhang et al. [26] designed an error fusion of multiple sparse autoencoders (EFMSAE) to monitor the health of a 3D printer in real-time. We can use the square prediction error (*SPE*) as an error fusion tool to fuse multiple SAEs, which positively affects feature extraction and health status detection. EFMSAE can check the health status of machinery, but it cannot predict health status. Inspired by the above discussion, this paper proposes a novel condition prediction method, named error fusion of hybrid neural networks (EFHNN), which combines the error fusion of multiple sparse auto-encoders with CNN for predicting the mechanical condition. First, in order to improve the accuracy of device state prediction, we calculate the square prediction error (*SPE*) by the error fusion method of multi-sparse self-encoder (EFMSAE) to extract the multi-feature information and obtain the trend curve representing the machine state. Then, we input the trend curve into the convolutional neural network for condition prediction. Thus, the main contributions of this paper include: (1) The square prediction error trend curve based on the error fusion of multi-sparse self-encoder model is acquired to denote the machine condition and a threshold control line is calculated to monitor the start time of mechanical failure. (2) Combining EFMSAE and CNN, an error fusion of hybrid neural networks (EFHNN) method is proposed for mechanical condition prediction, which improves the accuracy of fault prediction. Thus, a novel condition prediction method, named error fusion of hybrid neural networks (EFHNN), is proposed and the main contributions of this paper include: (1) The square prediction error trend curve based on the error fusion of multi-sparse self-encoder model is acquired to denote the machine condition and a threshold control line is calculated to monitor the start time of mechanical failure. (2) Combining EFMSAE and CNN is used for mechanical condition prediction, which improves the accuracy of fault prediction.

The rest of the paper is organized as follows. The second section introduces the theoretical basis of the error fusion of hybrid neural networks method. The third section introduces the bearing life test and the four-axis unmanned aerial vehicle (UAV) accelerated failure test. After that, we will discuss the experimental results in Section 4. Finally, we draw conclusions and future research directions in Section 5.

## 2. Methodology

The proposed error fusion of hybrid neural networks is described in this section. The based knowledge of the SAE and CNN method is introduced for raw data processing in the first and second parts. Subsequently, the detail techniques of the proposed EFHNN are described. Finally, the flow chart of the method proposed in this paper is introduced in the fourth part.

### 2.1. Sparse Auto-Encoders

**Figure 1:**
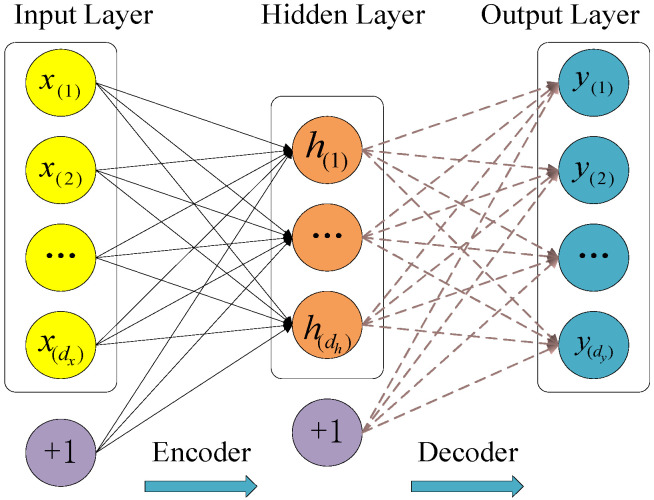
The structure of sparse auto-encoder.

An autoencoder (AE) is an unsupervised hidden layer neural network that automatically learns features from original data by minimizing reconstruction errors [27]. Sparse automatic encoding (SAE) is a hierarchical deep neural network structure composed of multiple layers of AEs. The goal of an AE is to reconstruct the original input as accurately as possible in the output layer [28]. The SAE method is used to learn features from the data set, and then multiple SAEs are error-fused to obtain the squared prediction error (*SPE*) trend curve. When the number of input neurons is greater than the number of output neurons, SAE will automatically complete the process of dimensionality reduction, that is, compress the data in the hidden layer and decompress them in the output layer. The model structure is shown in Figure 1.

While the sparse autoencoder reduces the dimensionality of the data, this method can ensure that the input and output values are as similar as possible. However, there are still some differences between input and output, which can be used for computing the square prediction error. It should be noted that the input and output have the same dimensions based on SAE in each layer, which is important for square prediction error. Thus, we used the SAE model to reduce the dimensionality of the data and compute the square prediction error. The characteristic curves extracted by traditional SAE and other algorithms are independent of each other, but the EFMSAE algorithm can fuse the characteristics of multiple channels to obtain a more accurate trend curve and threshold control line, which is convenient for monitoring the changing trend of the system.

The sparse restriction mechanism in SAE acts on the hidden layer to control the number of “active” neurons. The Sigmoid function is used as the activation function of the network, and its range is (0, 1), expressed as:

(1) $$D_{i+1}=\sigma \left(D_{i}\right)=\frac{1}{1+exp(D_{i})}$$

In the above formula, ***D****_i_* and ***D***_*i*+1_, respectively, represent the *i*-th input layer and the *i*-th output layer.

Suppose the input of the *i*-th hidden layer is ***X***, *x_i_* ∈ R(m), where N is the total number of data sets, and m is the dimensionality of each data set. In the automatic encoding network, the activation function σ acts on the input layer, and the encoding operation is performed on it to obtain the hidden vector h of the hidden layer. The hidden vector is decoded in the output layer to obtain the output vector ***a***, which is expressed by the formula:

(2) $$h=\sigma (E_{i}X+b_{1})$$

(3) $$a=\sigma (D_{i}h+b_{2})$$

In Equation (2), ***E****_i_* represents the encoding of the hidden layer; ***D****_i_* denotes the decoding weight of the output layer; and *b*_1_ and *b*_2_ represent the deviation values of encoding and decoding, respectively. We use hj (*x*(*i*)) to represent the output of the *j*-th neuron in the hidden layer, and *x*(*i*) to represent the input of the *i*-th sample.

### 2.2. Convolutional Neural Networks Method

There are some bottlenecks in the development of the deep neural network, and convolutional neural networks (referred to as CNN) is one of the most successful special cases. Ehsan Hoseinzade et al. [29] suggested a CNN-based framework that can be applied to collect data from various sources, including different markets, to extract features for predicting the future of those markets. Roberto Rosas-Romero et al. [30] used CNN to predict seizures in fNIRS signals for obtaining good results in terms of time series prediction.

**Figure 2:**
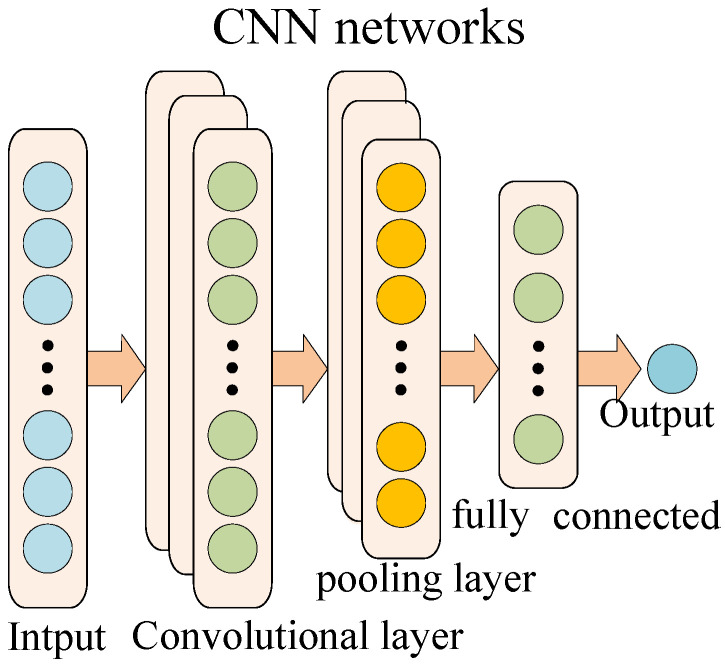
The structure of CNN.

A typical CNN mainly includes an input layer, a convolutional layer, a pooling layer, and a fully connected layer, as shown in Figure 2. The convolutional layer uses a convolutional kernel to perform convolution processing on the input data and output a feature map. Each convolutional kernel outputs a layer of neuron matrix, called a feature map. The process of convolution is described as follows:

(4) $$x_{i}^{l}=f(\sum_{i\in M_{j}}x_{i}^{l-1}*k_{ij}^{l}+b_{j}^{l})$$

where *l* represents the *l*-th convolutional layer, $x_{i}^{l}$ is the output of the *l*-th layer, $x_{i}^{l-1}$ is the input of the *l*-th layer, $k_{ij}^{l}$ represents the weight matrix, $b_{j}^{l}$ is the bias, *M_j_* is the *j*-th convolution regions of the *l* − **1** feature map, and *f*(•) is the activation function.

After the convolution operation, the activation function is also crucial. The activation function usually chooses ReLU, and its expression is:

(5) f(x)=max(0,x)

The pooling layer is usually used after the convolutional layer completes the convolution. It down samples the input feature vector through the pooling core, and further highlights the extracted features while achieving data dimensionality reduction. Pooling operations are generally divided into two types: max pooling and meaning pooling. The general expression of pooling is:

(6) $$x_{i+1}=f(\beta *down(x_{i})+b)$$

where *x_i_* represents the input, *down*( ) is the pooling function, ***β*** is the multiplicative bias, and *b* is the additive bias.

Since the vibration signal is input to the EFMSAE model, the output is an *SPE* curve fused with multi-channel errors, which is a one-dimensional time series. Therefore, we choose a one-dimensional convolutional neural network to predict the *SPE* value of the system. The convolution kernel of the CNN network is 2 × 2, the pooling process uses the maximum pooling, and the activation function selects the ReLU activation function.

### 2.3. Error Fusion of Hybrid Neural Networks

The initial goal of the autoencoder is to reduce the dimensionality. Generally, the number of nodes in the input layer is greater than the number of nodes in the hidden layer. We can also make the number of nodes in the hidden layer more excellent than the input layer’s number. However, it is difficult for the autoencoder to learn the sample features independently, and it is necessary to add a certain sparsity limit to the nodes in the hidden layer. Sparse auto-encoding is obtained by adding constraints based on the auto-encoder, suppressing most of the neurons in the hidden layer, and only activating a small part of the neurons to learn sample features. This method can ensure that the input and output values are similar, and the residual matrix can also be defined.

We use the deviation value to fuse multiple SAEs together to obtain the square prediction error (*SPE*) value and the *SPE* trend curve. On the basis of Equation (3), the reconstruction vector is ***A*** = {a1, a2,…, ai,…, aN−1, aN}, ai ∈ R(m). The sparse encoder needs to ensure that the dimensions of the output data set and the input data set are the same, so the residual matrix ***R*** can be expressed by ***X*** and ***A***, and expressed as:

(7) X=A+R

We use *k* to represent the sensor number, that is, *k* = 1, 2, 3, …, H. Then, the statistics of the *SPE* can be obtained from the error function, and the formula is:

(8) $$SPE_{k}={\left(x_{k}-a_{k}x_{k}\right)}^{T}\left(x_{k}-a_{k}x_{k}\right)$$

In order to estimate the threshold at any time, the control limit needs to be calculated in the *SPE*. The distribution of the *SPE* can be defined as:

(9) $$SPE_{\alpha }=\left(\frac{v}{2h}\right)\chi_{2h^{2}/v}^{2}\left(\alpha \right)$$

In Equation (9), α represents the confidence of the chi-square distribution variable, generally 0.05; h represents the average value of the *SPE* in the data set; *v* represents the variance value of the *SPE* in the data set; $2h^{2}/v$ represents the degree of freedom of the *SPE* in the data set. *h* and *v* are expressed as:

(10) $$h=\frac{1}{K}\sum_{i=1}^{K}\left(x_{i}-\hat{x}\right)$$

(11) $$v=\frac{1}{m}{\sum_{i=1}^{m}\left(h_{i}-\hat{h}\right)}^{2}$$

where *K* represents the *k*-th batch of the normal process, and *m* represents the dimension of the data set.

When the machine is working normally, the *SPE* values represented by the error function are very low. When an abnormal situation occurs, the error value will suddenly increase, causing the instantaneous *SPE_k_* value to exceed the threshold. Therefore, the evaluation criteria for mechanical failure can be expressed as:

(12) $$\left\{\begin{array}{l} SPE_{k}\leq SPE_{\alpha }\Rightarrow \mathrm{healty}\\ SPE_{k}\geq SPE_{\alpha }\Rightarrow \mathrm{faulty} \end{array}\right.$$

### 2.4. Overview of the Proposed Algorithm

**Figure 3:**
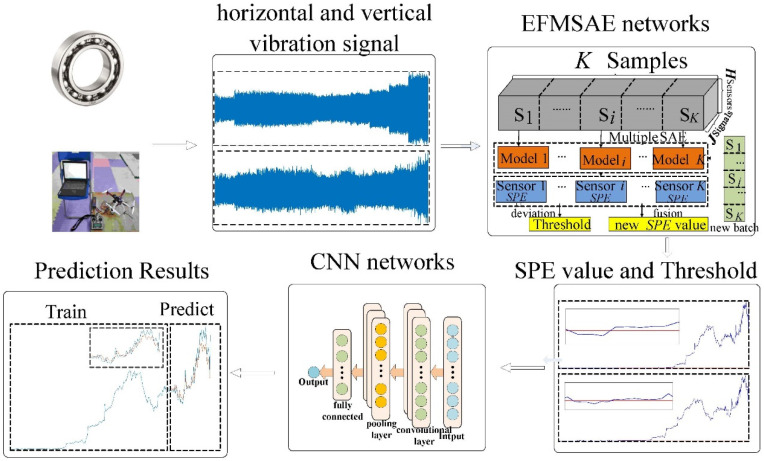
The overall proposed EFHNN structure.

The whole algorithm is divided into two parts: EFMSAE and CNN. First, EFMSAE obtains feature extraction and the *SPE* trend curve from the sensors data that have been preprocessed. Secondly, the feature vector and trend curve of the CNN model are predicted and estimated. The overall proposed EFHNN structure is shown in Figure 3, and can be summarized as follows.

- Step 1. Collect the full life cycle data set based on different sensors released by Xi’an Jiaotong University and UAV propellers.
- Step 2. Extract multi-feature sequence representation of unlabeled data by multiple SAEs.
- Step 3. On the basis of conventional data batch processing, the threshold line is estimated according to the conventional data batch, and the *SPE* value is calculated according to the test data.
- Step 4. Combine the *SPE* value of the multi-channel sensor to obtain the trend curve of the system.
- Step 5. CNN is used to forecast the trend of time series with multi-feature fusion, and analyze the level of anomaly of its parts. End.

## 3. Experiments

This section may be divided by subheadings. It should provide a concise and precise description of the experimental results, their interpretation as well as the experimental conclusions that can be drawn.

### 3.1. The Toolbar and Its Menus

**Figure 4:**
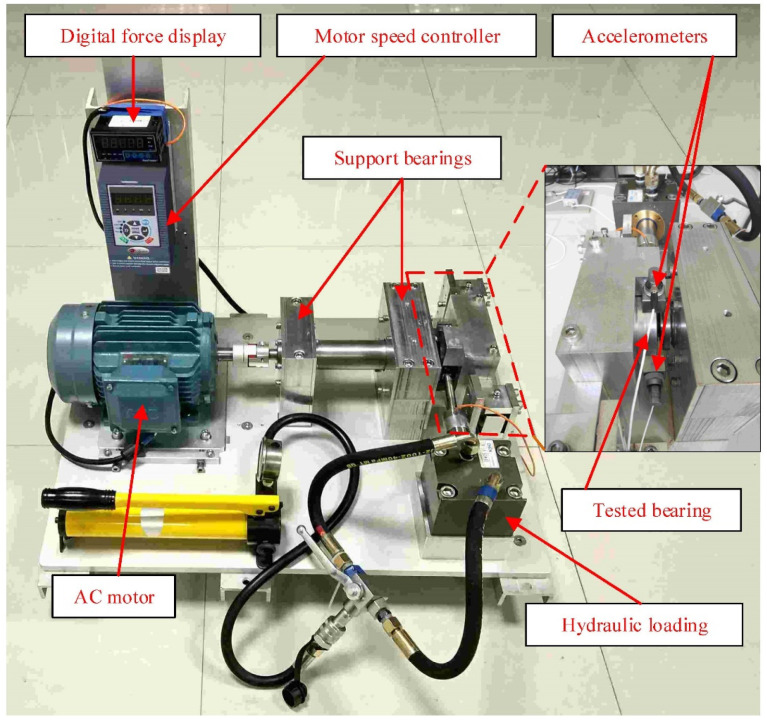
Bearing experimental setup.

We used the XJTU-SY bearing data set, which contains complete run-to-failure data of 15 rolling element bearings that were acquired by conducting many accelerated degradation experiments. These data sets are public and have a certain degree of persuasiveness for the verification algorithm. We used it to verify the algorithm and finally obtained good results. The bearing experimental setup is shown in Figure 4.

The platform can carry out accelerated degradation experiments of various rolling bearings or sliding bearings to obtain monitoring data of the bearings’ full life cycle. The tested bearing faults cover the outer ring, inner ring, cage, and rolling elements. The types of faults include outer ring wear, outer ring cracks, inner ring wear, cage fracture, etc. The bearing used in this experiment is LDK UER204 rolling bearings, as shown in Table 1.

The two acceleration sensors (PCB 352C33) are magnetically fixed in the horizontal and vertical directions of the bearing to be tested. This solution is used to obtain the complete life cycle vibration signal data of the bearing. In the experiment, a DT9837 portable dynamic signal collector was used to collect vibration signals. The sampling frequency is set to 25.6 kHz, the sampling interval is 1 min, and the duration of each sampling is 1.28 s.

In this data set, the vibration signals are stored in CSV format files and named in the order of sampling time. The first column represents horizontal vibration signals, and the second column represents vertical vibration signals.

### 3.2. Experiments in Unmanned Aerial Vehicle

**Figure 5:**
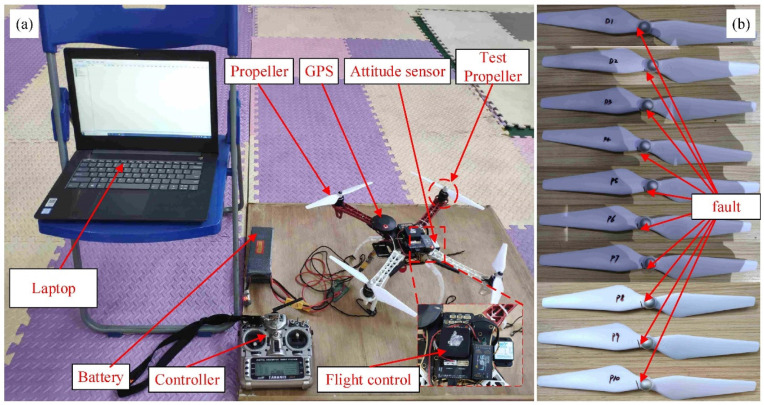
Unmanned aerial vehicle experimental setup: (**a**) The overall structure; (**b**) Crack fault condition.

This article also uses a four-axis unmanned aerial vehicle (UAV) as the experimental object to conduct a propeller accelerated destruction experiment. The experimental device is shown in Figure 5. In this experiment, the test object is a four-axis unmanned aerial vehicle (UAV), which consists of a flight controller (cube PIXHAWK2), GPS, propellers, and other components. There are two 16,000 mah batteries (3S 25C) connected in parallel to make sure that the experiment is carried out with sufficient motivation. The attitude sensor (BWT901CL) is installed on the UAV support board to transmit the data signal to the computer in real-time. We used wireless transmission to reduce interference and increase the reliability of the experiment. The attitude sensor can collect attitude data during UAV flight. The low-cost attitude sensor can acquire attitude data during the drone experiment. The sensor can work in a very high temperature fluctuation range, and the attitude data are composed of triaxial acceleration, triaxial angular velocity, triaxial magnetic field and triaxial angle signals. The measurement stability of the attitude sensor is 0.01°, the sampling frequency is 200 Hz, and the total acquisition time is set to 5 min.

In this experiment, the four-axis UAV flies under full load, that is, the four supporting legs of the UAV are connected to the board. At this time, the drone can only be hovering. The vertical distance between the bottom of the drone and the surface of the board is 160 mm. In this experiment, an acceleration failure experiment was performed at the position of the right front axle. The simulated fault location of the propeller root is 10 mm from the center of rotation. There are ten propellers with different degrees of damage, denoted as 1 mm, 2 mm, ..., and 10 mm, respectively, which are shown in Figure 5b.

## 4. Validation of the Proposed Algorithm

In this section, the accuracy of the proposed algorithm is verified and evaluated by analyzing the experimental results of rolling bearings and UAVs. The evaluation of the algorithm is mainly carried out from these several parameters: average absolute error (*MAE*), average absolute percentage error (*MAPE*), mean square error (*MSE*) and root mean square error (*RMSE*). When experimenting with rolling bearings and UAVs, we used the XJTU-SY rolling bearing component life test data set and UAV propeller acceleration failure data set published by Xi’an Jiaotong University. The bearing data points were divided into different data sets based on total samples, and the first five data sets obtained when the bearing works normally are used as the standard for subsequent evaluation. Then, the *SPE* value of the multi-channel sensor is obtained and CNN is used to forecast the trend of time series with multi-feature fusion.

Mean absolute error (*MAE*) refers to the average of the absolute value of the error between the predicted value and the observed value. The average absolute error can avoid the cancellation of the positive and negative errors, so it can better reflect the actual error size. We use Equation (13) to calculate, where n represents the number of samples, and *f_t_* and *y_t_* represent the variable values of the same phenomenon.

(13) $$MAE=\frac{1}{n}\sum_{t=1}^{n}\left|f_{t}-y_{t}\right|$$

Mean absolute percentage error (*MAPE*) is usually a statistical indicator that measures the accuracy of forecasts, such as time series forecasts. The smaller the value of *MAPE*, the higher the accuracy of the model. It is expressed by Equation (14), where *n* represents the number of samples, *y_t_* represents the observed value, and *f_t_* represents the predicted value.

(14) $$MAPE=\frac{100\%}{n}\sum_{t=1}^{n}\left|\frac{y_{t}-f_{t}}{y_{t}}\right|$$

The mean square error (*MSE*) is the average of the sum of squares of the difference between the predicted value and the observed value, and is often used as an indicator to measure the predicted result. We use Equation (15) to calculate the mean square error, where *n* represents the number of samples, *y_t_* represents the observed value, and *f_t_* represents the predicted value.

(15) $$MSE=\frac{1}{n}{\sum_{t=1}^{n}\left(f_{t}-y_{t}\right)}^{2}$$

The root mean square error (*RMSE*) is based on the mean square error, and it can be used to measure the deviation between the predicted value and the observed value. The graph can be calculated with Equation (16), where n represents the number of samples, *y_t_* represents the observed value, and *f_t_* represents the predicted value.

(16) $$RMSE=\sqrt{MSE}=\sqrt{\frac{1}{n}{\sum_{t=1}^{n}\left(f_{t}-y_{t}\right)}^{2}}$$

### 4.1. Validations Using Rolling Bearing Data

This part mainly introduces the data preprocessing and parameter setting of the algorithm, and predicts, analyzes and discusses the failure time series from different threshold starting points.

#### 4.1.1. Data Preprocess and Parameter Set

The XJTU-SY bearing life test data set published by Xi’an Jiaotong University is used for auxiliary verification. We used bearing 2_3, bearing 2_5 and bearing 3_4 to conduct experiments on multiple data sets. Bearing 2_3 and bearing 2_5 are under the conditions of a load of 11 KN and a speed of 37.5 Hz, while the operating condition of bearing 3_4 is 10 KN load with 40 Hz speed. The *SPE* value of the training sample can be obtained by Equations (8) and (9), and the corresponding *SPE*α and *SPE*_*k*_ values can also be obtained. Then, the characteristic curve and the comprehensive trend curve of the *SPE* value corresponding to the two channels can be obtained.

Rolling bearings will have different types of failures under different working conditions, which are mainly divided into performance degradation failures and sudden failures [31]. The bearing performance degradation failure can be divided into three situations: (1) After exceeding the threshold line, the value rises slowly (for example, bearing 2_3); (2) After exceeding the threshold line, a small part of the value suddenly rises and then tends to stabilize (for example, bearing 2_5); (3) After exceeding the threshold line, the value continues to rise (for example, bearing 3_4). We noted that the total samples for bearing 2_3, bearing 2_5 and bearing 3_4 are 533, 339 and 1515, respectively. The final failure position for these bearings is cage, outer ring and inner ring. The data set was preprocessed, and the total data points were collected from the normal operation of the rolling bearing to the failure, including horizontal and vertical vibration signals. These data points were divided into different data sets based on total samples, and the first five data sets obtained when the bearing works normally are used as the standard for subsequent evaluation. Through the EFMSAE model, the obtained data set has the characteristics of low dimensionality and obvious characteristics.

**Figure 6:**
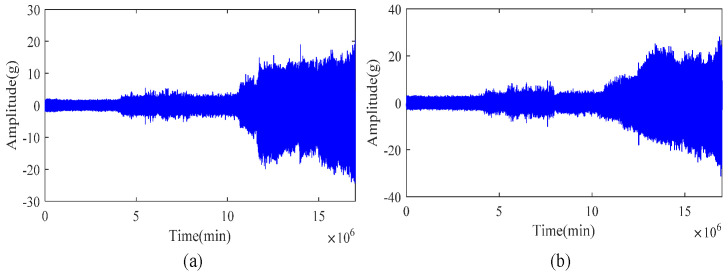
Typical bearing performance degradation gradients over the full life cycle of time domain: (**a**) horizontal vibration signal; (**b**) vertical vibration signal.

Figure 6 shows the performance degradation gradient during the entire life cycle of the bearing from the beginning to the failure, where Figure 6a shows the time domain horizontal vibration signal, and Figure 6b shows the time domain vertical vibration signal. It can be seen from the figure that the life cycle of a bearing includes two different phases, namely the normal working phase and the degraded phase. In the normal working phase, the vibration signal only exhibits low-level random fluctuations. In the degradation stage, the vibration signal has an obvious upward trend, which shows that there is a wealth of bearing degradation information in this stage.

Rolling bearings are in a stable state for most of the entire working cycle, and it is not advisable to have stable state training to predict fluctuating fault states. Therefore, it is necessary to select the parts with obvious fluctuations beyond the threshold line and input them into the CNN model. The first 80% of samples are used as training samples, and the last 20% of samples are used as subsequent test samples. The model takes a time step between every two predictions, and updates the network state during each prediction. In the rolling bearing 2_3, the CNN layer has 200 hidden units, and 100 rounds of training have been carried out. The initial learning rate is 0.005. After 60 rounds of training, the learning rate is multiplied by a coefficient of 0.2 to decrease. In addition, to prevent the gradient from exploding, the gradient threshold is set to 1.

#### 4.1.2. Analysis and Discussion on Experimental Results of Bearing Operation

**Figure 7:**
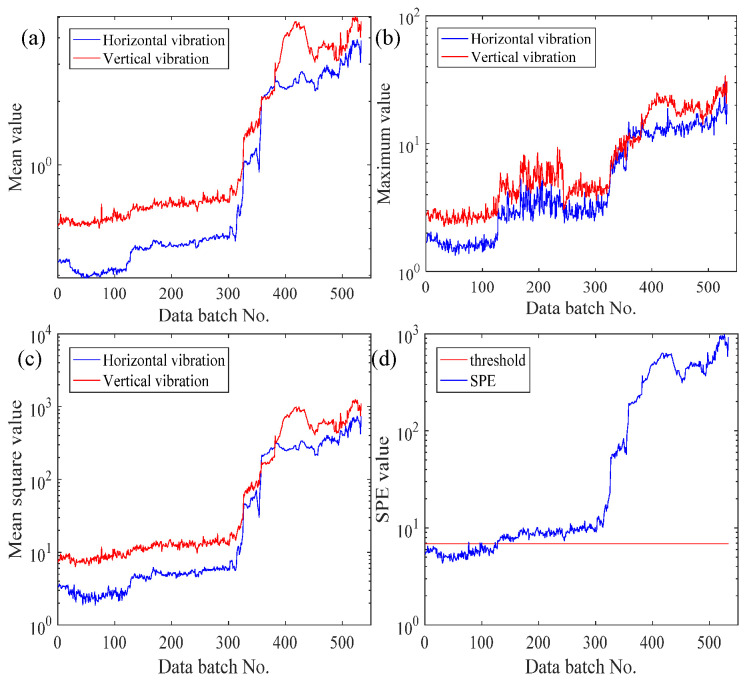
The curves of different data processing methods of Bearing 2_3: (**a**) Mean value; (**b**) Maximum value; (**c**) Mean square value; (**d**) *SPE* value.

In order to show the benefits of the EFMSAE method in failure time series prediction, we use the average, maximum, mean square and *SPE* value of each batch of 2_3 bearings for analysis. Figure 7a–d are the mean value curve of horizontal and vertical vibration signals of the vibration sensor, the maximum value curve of the vibration sensor, the mean square value curve of the vibration sensor, and the fusion curve of *SPE* vibration signal based on horizontal and vertical directions, respectively.

Based on Professor Lei’s interpretation [31], it can be concluded that bearing 2_3′s failure position is the cage. It can be understood from Figure 7 that the vibration signal in the following four pictures rises slowly as the batch increases, and after the 300th batch, it rises sharply and drops around the 400th batch. This is because the bearings were worn out in the 128th batch, and vibration was positively correlated with wear. As the stress in the damaged area increased, repairable damage (approximately 40th batch) was caused because the material near the damaged area of the bearing filled it. Increased stress in the damaged area can cause devastating damage. The analysis obtained by observing the four graphs of average value, maximum value, average variance and *SPE* value is as follows.

The two local maximum values of the vertical vibration signal of the average curve are almost equal, which is not consistent with the actual fault condition, so its performance characteristics are not satisfied. Regardless of whether it is a vertical signal or a horizontal signal in the maximum curve, the adjacent batches fluctuate greatly, which reduces the observability of the graph and causes great trouble in data processing. However, the mean square curve does not have the problems of the above two curves, and can better represent the changing trends of the two vibration signals.

Therefore, compared to the single-channel vibration signal of the mean square curve, the signal of integrating the data of the two channels into one *SPE* curve based on the mean square curve is more complete, more reliable, and less difficult for data processing. The above proves the superiority of the *SPE* value obtained by EFMSAE. Putting it and the threshold line of the system failure in a graph for analysis, it can be known that the *SPE* value has exceeded the threshold since the 128th batch. It can be considered that the rolling bearing 2_3 may have been invalid in the 128th batch.

**Figure 8:**
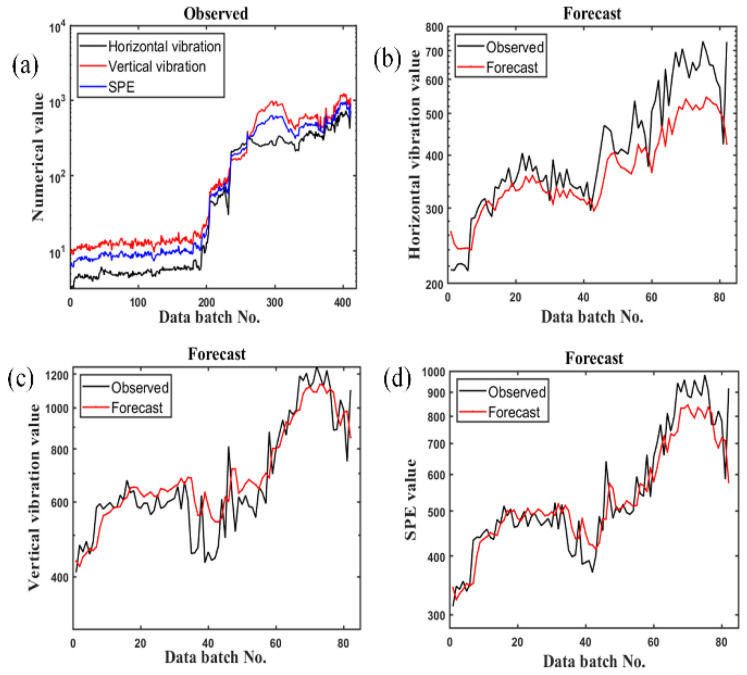
The curve of the last 20% observed value and the forecast value of Bearing 2_3: (**a**) All original value; (**b**) horizontal vibration value; (**c**) vertical vibration value; (**d**) *SPE* value.

EFMASAE is used to process the data set. Figure 8 shows the analysis of the data after the rolling bearing 2_3 was invalid (128th batch). Using the CNN model, the ratio of training to testing samples is 8 to 2. Figure 8a–d are the observed values of the mean square error curve, the observed and predicted values of the horizontal vibration data, the observed and predicted values of the vertical vibration data, and the curves of predicted and observed *SPE* values, respectively. The predicted value of the horizontal signal has a good forecast trend in the first half but obviously differs from the observed value in the second half. The predicted value of the vertical signal can roughly reflect the trend of the observed value, but the effect is still not satisfactory. However, the *SPE* curve after the dual-channel fusion can not only reflect the trend of the observed value, but it also has better indicators in all aspects than Figure 8b,c, which will be described below. We use mean square error (*MSE*), root mean square error (*RMSE*), mean absolute error (*MAE*) and mean absolute percentage error (*MAPE*) to evaluate the performance of the algorithm, which is more convincing than directly observing the data.

In order to avoid the instability of a single experiment, we perform five times for each group of experiments and evaluate the performance of the algorithm with an average value, as shown in Table 2.

Table 2 shows that the *MSE* value, *RMSE* value, *MAE* value and *MAPE* value of the *SPE* curve are all lower than horizontal and vertical vibration signal curves. Based on various evaluation criteria, it can be proved that the prediction curve of the *SPE* value is better than the prediction curve of the mean square deviation of the single channel, and it has better prediction effect.

**Figure 9:**
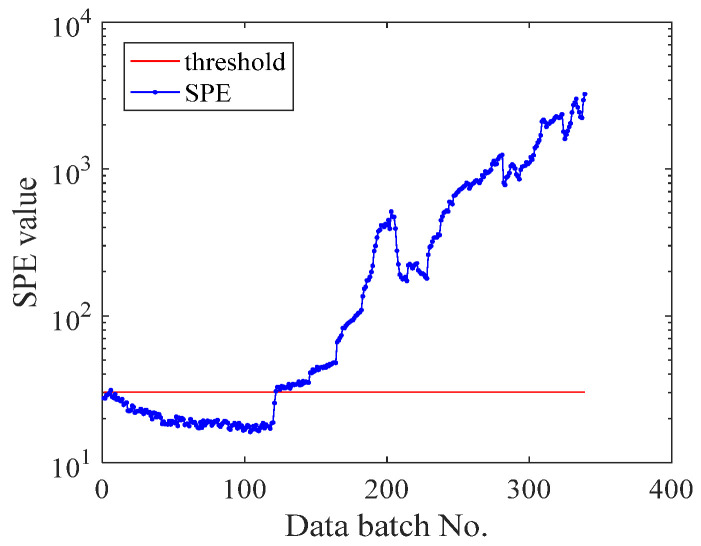
The *SPE* curve of the last 20% observed value and the forecast value of Bearing 2_5.

**Figure 10:**
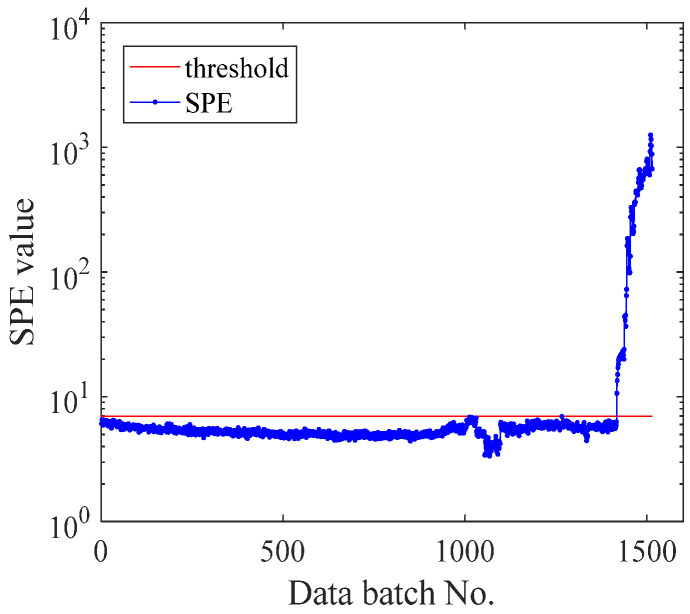
The *SPE* curve of the last 20% observed value and the forecast value of Bearing 3_4.

The above experimental data show that the *SPE* curve can express more bearing information than others, so the *SPE* curve is more qualified as the evaluation standard of bearing time series prediction. The *SPE* curves of three different types of bearings are, respectively, analyzed below. The *SPE* curves of bearing 2_3, bearing 2_5, and bearing 3_4 are vividly shown in Figure 8d, Figure 9 and Figure 10, respectively. As shown in Figure 7d, bearing 2_3 begins to fail at the 128th batch of the *SPE* curve, where its *SPE* value exceeds the threshold line. Then, its *SPE* curve rises exceptionally slowly. Bearing 2_5′s failure occurred in the 122nd batch of the *SPE* curve, as shown in Figure 9. Since the bearing’s failure position is the outer ring, bearing 2_5 will fail faster than bearing 2_3. With the continuous effect of stress on the failure position, bearing 2_5 will eventually fail completely. As shown in Figure 10, the failure of bearing 3_4 begins in the 1418th batch of the *SPE* curve. As the bearing’s failure position is the inner ring, the *SPE* curve rises rapidly after exceeding the threshold line, which is different from the other two bearings. The prediction effect shown by the *SPE* curve is consistent with the fault trend of three different types of bearings, and the threshold line obtained by EFMSAE can reasonably predict the time of bearing fault. Therefore, the *SPE* curve can accurately express the bearing fault information and fully meet the fault prediction and evaluation curve criteria.

**Figure 11:**
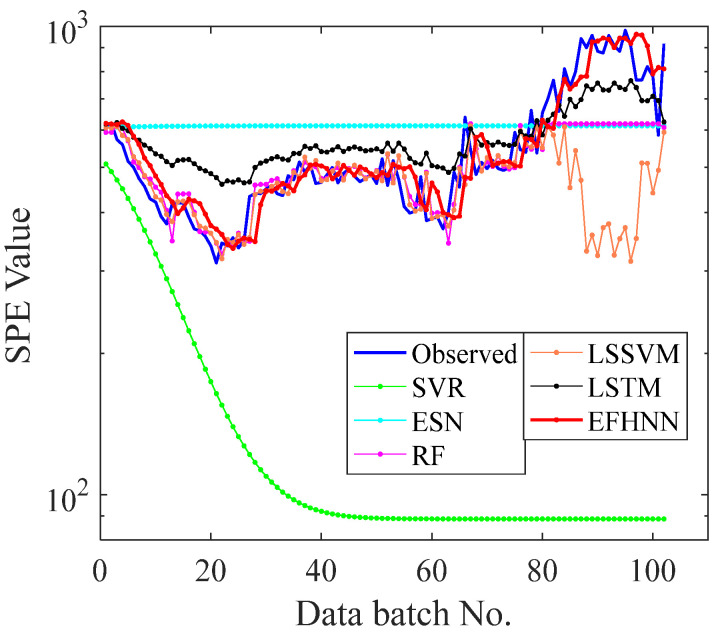
The curves of observed and forecast values of Bearing 2_3 EFHNN.

**Figure 12:**
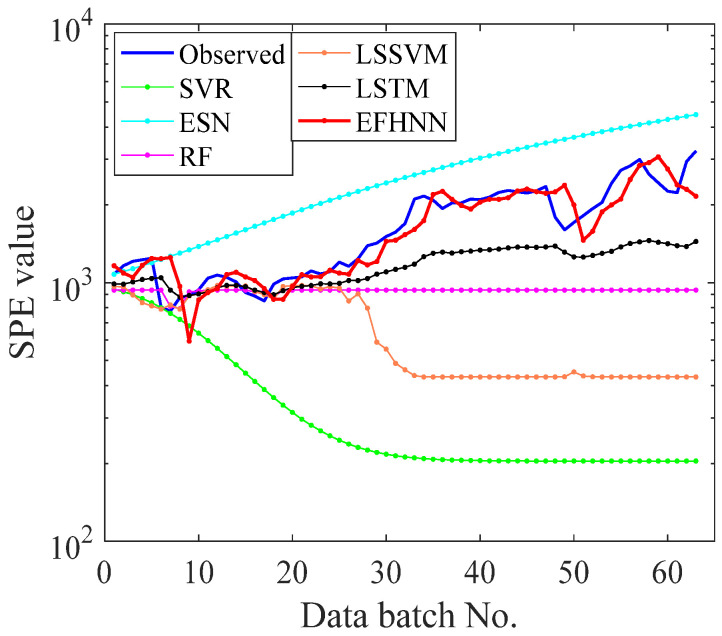
The curves of observed and forecast values of Bearing 2_5.

**Figure 13:**
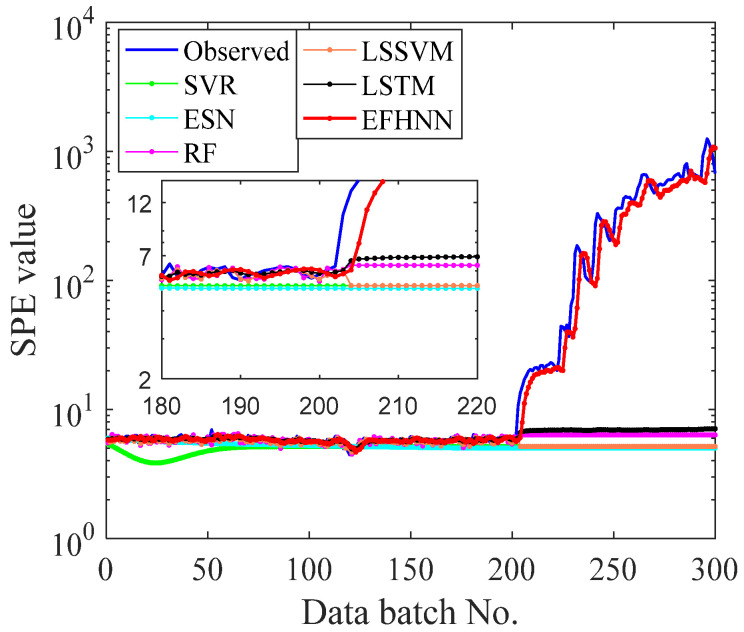
The curves of observed and forecast values of Bearing 3_4.

In this paper, five other prediction methods were introduced for experimental comparison to verify the CNN model’s prediction performance and advantages. To ensure the experimental reliability and fairness, all methods used the same original data set without preprocessing. The training set and test set were divided in the same proportion, and the independent experiment was repeated five times. Detailed data of evaluation indicators are shown in Table 3. To analyze the experimental prediction results vividly, all method prediction curves of bearing 2_3, bearing 2_5, and bearing 3_4 are visualized in Figure 11, Figure 12 and Figure 13, respectively.

It can be seen from Table 3 that the values of three evaluation indexes of the CNN model are far less than those of other models in three different decay type bearing experiments, indicating that the prediction curve of the CNN model is closer to the *SPE* curve. The CNN model is superior to other models in fault trend prediction of rolling bearing. According to the visualization figure, the prediction function of SVR and ESN completely fails when they encounter a small kurtosis at the beginning of the prediction of the three kinds of bearings, while the prediction function of RF and LSSVM fails when they encounter a large kurtosis change in the 80th batch of bearing 2_3, the 28th batch of bearing 2_5, and the 200th batch of bearing 3_4, respectively. The LSTM model predicts better performance than the above four methods, and the prediction effect is not good but failure at about the 80th batch of bearing 2_3 and the 28th batch of bearing 2_5, respectively. Except for the CNN model, the other models cannot effectively predict the failure curve of bearing 3_4. However, the prediction curve of the CNN model can follow the change in the *SPE* curve in three different cases of bearing failure and accurately predict the health change trend of bearing. The prediction performance of the EFMSAE method combined with the CNN model for three different types of bearing failure is analyzed from the perspective of different evaluation indexes and visualization graphs. Compared with other methods, the experimental results show that the proposed method is effective and stable in the prediction of rolling bearing fault time series.

### 4.2. Validations Using Unmanned Aerial Vehicle Data

#### 4.2.1. Data Preprocess and Parameter Set

In this experiment, the attitude sensor was used as the original signal input. The data set was preprocessed and 1,660,164 data points were collected. Then, we used the acceleration sensor to analyze the data, the sampling interval was 30 s, and we divided the data points into 3 × 15,000 × 110 data sets. In the same way, the first 3 × 15,000 × 5 data sets in the normal state of the drone are used as the standard for subsequent evaluation.

**Figure 14:**
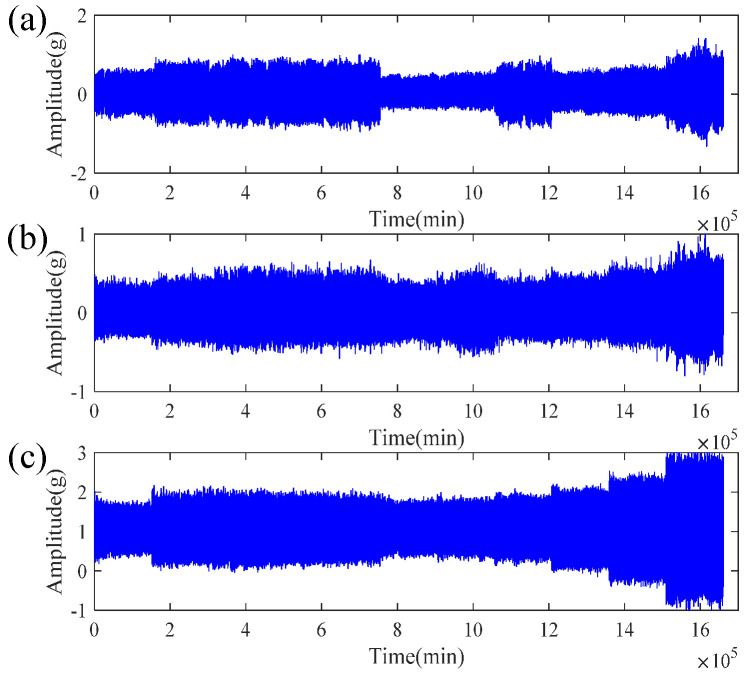
The original time domain signal of the UAV propeller: (**a**) *X*-axis acceleration signal; (**b**) *Y*-axis acceleration signal; (**c**) *Z*-axis acceleration signal.

By processing the data through the EFMSAE model, the characteristic curve of the three channels and the trend curve of the *SPE* value can be obtained—the sizes are 3 × 110 and 1 × 110, respectively. The three vibration signals measured on the propeller using the acceleration sensor are shown in Figure 14.

#### 4.2.2. Analysis and Discussion on Experimental Results of Unmanned Aerial Vehicle

**Figure 15:**
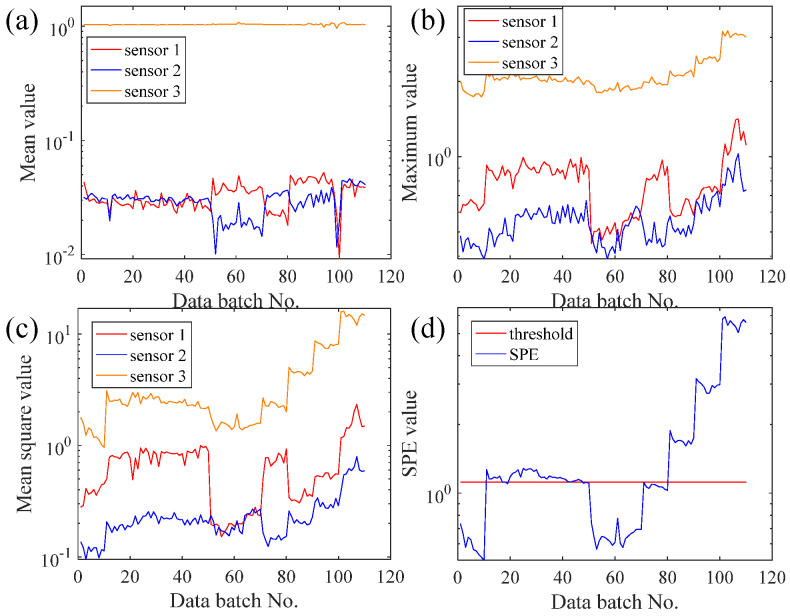
The curves of different data processing methods of UAV: (**a**) Mean value; (**b**) Maximum value; (**c**) Mean square value; (**d**) *SPE* value.

To further verify the EFMSAE model’s performance, the average value, maximum value, mean square value, and *SPE* value of each batch of UAV propellers were taken for a comparison experiment, just like the bearing experiment above. The average, maximum, and mean square error curves of the acceleration sensor in three directions, X, Y, and Z, are shown in Figure 15a–c, respectively. Moreover, the *SPE* value curve combining the information of three directions is shown in Figure 15d. As shown in the visualization diagram, the average value curve’s characteristic information is not significant enough. The feature information expressed by the three channels of the maximum curve is different. The feature information represented by the second curve in the mean square value curve is not changed. However, the *SPE* curve is a curve which integrates the information of three sensors, and the feature information expressed is very diverse and rich. It can be seen from Figure 15d that the *SPE* value exceeds the threshold control line at the 11th batch, which means that the UAV propeller starts to fail at this point. Due to the blade vibration fatigue crack after UAV propeller crack failure, the *SPE* curve will drop in the 51st and 70th batches. With the crack propagation, different blades’ surface shape will be different due to different blade thicknesses. The overall spread rate of the UAV propeller first increases then decreases, and then increases. All these indicate that the *SPE* curve can represent the UAV propeller performance’s changing trend in the whole system.

**Figure 16:**
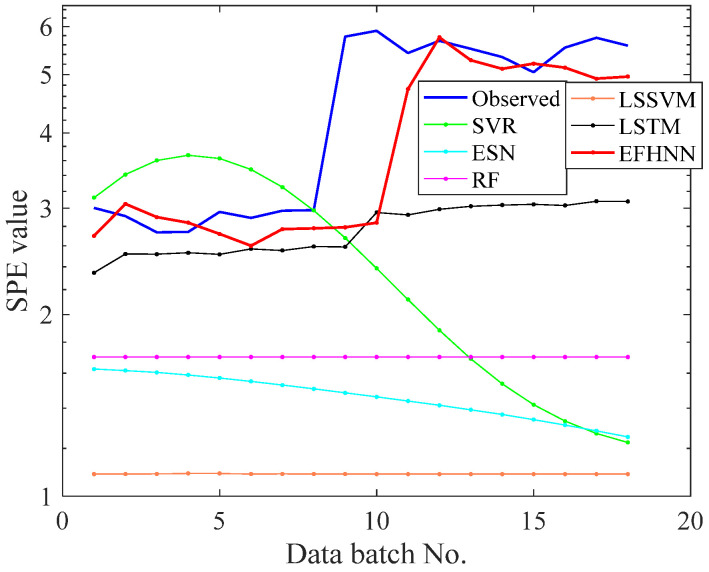
The curves of observed and forecast values of Bearing 2_3.

To further verify the CNN model’s prediction performance, the VAR data set was used for prediction experiments. The training set ratio to test set and other parameters’ setting were the same as the above bearing experiment. Table 4 shows the average evaluation values of five repeated independent experiments, and the prediction curves of all methods are visualized in Figure 16.

As shown in Figure 16, in the face of dramatic changes in propeller failure of the UAV (*SPE* curve suddenly rises, with an extensive change range), the prediction function of SVR, ESN, RF, and LSSVM in the 11th batch is out of service. The prediction effect of LSTM is deplorable. Only the CNN model can overcome this change, and its prediction curve is better than other models. It can also be seen from the evaluation indexes in Table 4 that the three index values of the CNN model are smaller than those of other models. That means that the CNN model’s prediction curve is the closest to the *SPE* curve, indicating the CNN model’s accurate prediction performance. The experimental results show that the CNN model is better than the other models and has good prediction performance in UAV propeller health prediction, which further proves that the proposed method has substantial accuracy and stability for time series prediction. The experimental results show that the CNN model is better than the other models and has good prediction performance in UAV propeller health prediction. It also further proves that the proposed method has vital accuracy and stability in predicting mechanical time series.

## 5. Conclusions

In this paper, an EFHNN method for predicting time series of mechanical property degradation was presented. This method combined the advantages of the EFMSAE neural network and CNN. Firstly, the EFMSAE neural network presented various features from the input vibration signals and obtained the *SPE* trend curve expressing the mechanical properties and the threshold line representing the performance degradation. The CNN model then predicted the mechanical performance state according to the original data exceeding the threshold line. The method was verified by three different types of rolling bearing and artificial accelerated aging test data, and compared with other advanced prediction methods. The complete experimental results show that the proposed method can effectively and stably predict the time series performance of mechanical failure for various data sets, and is superior to other methods. Moreover, this method can also be extended to predicting key mechanical states such as gears, shafts, propellers, and cutters.

## Figures and Tables

**Table 1:** Bearing accelerated life test conditions.

Operating Condition	Radial Force (kN)	Rotating Speed (rpm)	Bearing Dataset
1	11	2250	Bearing2_3 Bearing2_5
2	10	2400	Bearing3_4

**Table 2:** Performance comparison of curves before and after fusion of Bearing 2_3.

Curve	*MSE*	*RMSE*	*MAE*	*MAPE*
H-mean square value	9.80 × 10^3^	98.50	70.05	13.79
V-mean square value	9.73 × 10^3^	98.61	72.81	10.52
*SPE* value	7.00 × 10^3^	82.42	59.35	9.71

**Table 3:** Comparison of predicted trends with different methods and threshold numbers.

Bearing Type	Method	*RMSE*	*MAE*	*MAPE*
Bearing 2_3	SVR	449.10	389.23	68.58
	ESN	182.60	161.54	32.74
	RF	115.53	72.23	10.89
	LSSVM	197.66	105.22	14.48
	LSTM [32]	105.67	90.94	17.87
	EFHNN	66.83	49.23	9.17
Bearing 2_5	SVR	1505.93	1255.15	68.11
	ESN	1045.14	894.17	54.91
	RF	950.96	716.35	35.22
	LSSVM	1296.33	974.10	47.27
	LSTM [32]	673.44	503.12	25.03
	EFHNN	299.86	212.78	12.92
Bearing 3_4	SVR	268.87	117.70	40.11
	ESN	268.93	117.51	36.21
	RF	268.36	116.91	32.65
	LSSVM	268.87	117.27	32.87
	LSTM [32]	268.02	116.63	31.83
	EFHNN	72.10	24.73	15.72

**Table 4:** Comparison of predicted trends with different methods and threshold numbers.

Method	*RMSE*	*MAE*	*MAPE*
SVR	2.67	2.06	41.77
ESN	3.04	2.64	58.07
RF	2.80	2.43	53.27
LSSVM	3.34	3.02	68.89
LSTM [32]	2.26	1.92	41.54
EFHNN	**1.07**	**0.61**	**12.15**

## Data Availability

Not applicable.
